# Ceftazidime-avibactam and aztreonam as a therapeutic alternative in two episodes of bacteremia due to *Stenotrophomonas maltophilia* after allogeneic hematopoietic stem cell transplantation: case report

**DOI:** 10.17843/rpmesp.2026.431.15393.

**Published:** 2026-03-02

**Authors:** Wilder Bolaños-Vargas, Cesar Copaja-Corzo, Ivan Fernandez Vertiz, Alfredo Wong-Chang, Miriam Rut Baldeon Laureano

**Affiliations:** 1 Pharmacy Department, Hospital Nacional Edgardo Rebagliati Martins, Lima, Peru.; 2 Health Evidence Generation and Synthesis Research Unit, Universidad San Ignacio de Loyola, Lima, Peru.; 3 Infectious Diseases Service, Hospital Nacional Edgardo Rebagliati Martins, Lima, Peru.; 4 Hematology Department, Special Hematology Service, Hospital Nacional Edgardo Rebagliati Martins, Lima, Peru.; 5 Hospital Nacional Edgardo Rebagliati Martins, Lima, Peru.

**Keywords:** Stenotrophomonas maltophilia, Hematopoietic Stem Cell Transplantation, Ceftazidime-avibactam, Aztreonam, Febrile Neutropenia

## Abstract

Infection due to *Stenotrophomonas maltophilia* represents a therapeutic challenge in immunocompromised patients, especially during hematopoietic recovery after hematopoietic stem cell transplantation (HSCT). The use of trimethoprim-sulfamethoxazole (TMP-SMX), the first-line antimicrobial, is contraindicated due to its hematological toxicity. We present the case of a patient undergoing haploidentical HSCT who developed two episodes of bacteremia due to *S. maltophilia* on days +20 and +49. Given the contraindication of TMP-SMX due to severe myelosuppression, the combination of ceftazidime-avibactam and aztreonam was used as an alternative treatment, achieving clinical resolution in both episodes. The patient remained stable until day +100; however, he experienced graft failure and died on day +107 due to infection with *Pseudomonas aeruginosa* producing VIM-type carbapenemase. This case shows that the use of ceftazidime-avibactam plus aztreonam can be useful against *S. maltophilia* infections in post-HSCT patients with contraindications for the use of TMP-SMX.

## INTRODUCTION

*Stenotrophomonas maltophilia* (*S. maltophilia*) is a non-fermenting Gram-negative bacillus recognized as an emerging opportunistic pathogen, particularly in immunocompromised patients, such as those undergoing allogeneic hematopoietic stem cell transplantation (HSCT) ^1^. In this context, profound and sustained immunosuppression favors both colonization and infection by environmental microorganisms such as *S. maltophilia*, which is capable of producing severe invasive infections, including pneumonia, persistent bacteremia, and sepsis ^2^^,^^3^. Its presence is associated with high morbidity and mortality in this group of patients, underscoring the importance of effective therapeutic strategies ^4^.

One of the main challenges in treating *S. maltophilia* infections lies in its intrinsic antimicrobial resistance profile, mediated by multiple mechanisms such as efflux pumps, alterations in outer membrane permeability, and the production of two chromosomal β-lactamases ^5^. The L1 β-lactamase is a metallo-beta-lactamase (MBL) that hydrolyzes penicillins, cephalosporins, and carbapenems, although it does not affect aztreonam. For its part, L2 is a serine β-lactamase with the capacity to inactivate extended-spectrum cephalosporins and aztreonam, but it is susceptible to inhibition by avibactam ^6^. This combination of mechanisms significantly reduces the activity of most conventional β-lactams against this pathogen ^7^. Despite this, trimethoprim-sulfamethoxazole (TMP-SMX) remains the treatment of choice ^8^.

However, in transplant patients with severe myelosuppression, the use of TMP-SMX may be contraindicated due to its hematological toxicity, including neutropenia and thrombocytopenia. In such clinical scenarios, it becomes necessary to resort to alternative therapies that preserve antimicrobial activity without compromising hematopoietic recovery ^9^^,^^10^. The combination of ceftazidime-avibactam (CZA) with aztreonam (ATM) has emerged as a promising therapeutic strategy against *S. maltophilia*, particularly in contexts of resistance or contraindication to TMP-SMX ^11^. This combination acts synergistically on the bacteria’s intrinsic enzymatic resistance mechanisms ^12^. Although clinical data on the combined use of CZA and ATM are still limited and come mostly from observational studies and case reports, in vitro evidence indicates that the mimetic formulation aztreonam-avibactam exhibits activity against approximately 92% of *S. maltophilia* isolates ^13^. Therefore, this combination is considered a valid therapeutic option in complex clinical scenarios, despite the lack of controlled studies.

In this context, we present the case of a patient undergoing HSCT, with an absolute contraindication for TMP-SMX, who developed two episodes of bacteremia due to *S. maltophilia* and was successfully treated with the combination of CZA and ATM. The objective of this report is to provide clinical evidence demonstrating the need to evaluate the use of CZA and ATM as a therapeutic alternative in post-HSCT patients during the phase of maximum marrow suppression (nadir) and hematopoietic engraftment, a scenario characterized by severe immunosuppression and limited antimicrobial options against *S. maltophilia*.

## CLINICAL CASE

A 36-year-old male with a diagnosis of myelodysplastic syndrome (MDS) with associated bone marrow fibrosis, classified as intermediate risk according to the International Prognostic Scoring System - Revised (IPSS-R) score ^3^. Due to his chronic transfusion requirement and the absence of an HLA-identical donor, a haploidentical HSCT with myeloablative conditioning was decided. The conditioning regimen included intravenous busulfan with a target area under the curve (AUC24h) of 4500 µmol·min and fludarabine with a target AUC24h of 5 mg·h/L (administered between days -6 and -2). On day 0, the infusion of hematopoietic stem cells (CD34+) was performed, followed by graft-versus-host disease (GVHD) prophylaxis with post-HSCT cyclophosphamide (50 mg/kg on days +3 and +4), and subsequently tacrolimus (0.075 mg/kg) plus mycophenolate mofetil (15 mg/kg/day) from day +5 to day +180.

### Intervention

From post-transplant day +1, the patient presented persistent fever (>38.3 °C) without an apparent clinical focus. Empirical treatment was started with piperacillin-tazobactam (4.5 g IV every 6 h). Given the lack of improvement, the regimen was escalated to meropenem (2 g IV every 8 h in a 3-hour extended infusion) starting on day +5. Persistent fever prompted the addition of vancomycin starting on day +10, with individualized adjustment via Bayesian modeling to reach an AUC/MIC between 400-600.

Between days +11 and +14, multiple peripheral and catheter blood cultures were negative. In the context of profound and prolonged neutropenia, intense immunosuppression, and lack of response to the broad-spectrum antibiotic regimen, invasive fungal infection (IFI) was suspected. Therefore, empirical antifungal treatment with caspofungin was established from day +13 (loading dose of 70 mg followed by 50 mg/day).

On day +20, the patient remained febrile despite being on treatment with meropenem, vancomycin, and caspofungin. Infection by multidrug-resistant Gram-negative bacilli was suspected; therefore, coverage with levofloxacin (750 mg PO every 24 h) and colistin (loading dose of 300 mg followed by 150 mg IV every 12 h) was added, and blood cultures were repeated. On day +23, the growth of Gram-negative bacilli was reported (blood culture from the central venous catheter [CVC] and peripheral taken on day +20), and given the lack of clinical improvement, meropenem and vancomycin were discontinued on day +25, and tigecycline 100 mg every 12h was started as part of a tiered strategy against multidrug-resistant pathogens.

On day +28, the presence of *S. maltophilia* was confirmed in CVC and peripheral blood cultures taken on day +20, showing sensitivity to levofloxacin and TMP-SMX (table 1). However, the use of TMP/SMX was ruled out due to its potential myelotoxicity, considering the patient’s state of marrow aplasia and the lack of transplant engraftment.

**Table 1:** Microbiological results of the first episode of bacteremia due to *Stenotrophomonas maltophilia*

Sample type	Date received	Isolate	Antimicrobial	MIC (µg/mL)	Interpretation
CVC Blood culture	04/12/25	*Stenotrophomonas maltophilia*	Levofloxacin	≤ 0.5	Sensitive
			Trimetoprim-sulfametoxazole	≤ 2/38	Sensitive
CVC Blood culture	04/10/25	*Stenotrophomonas maltophilia*	Levofloxacin	≤ 0.5	Sensitive
			Trimetoprim-sulfametoxazole	≤ 2/38	Sensitive
PB Blood culture	04/14/25	*Stenotrophomonas maltophilia*	Levofloxacin	≤ 0.5	Sensitive
			Trimetoprim-sulfametoxazole	≤ 2/38	Sensitive
CVC Blood culture	04/14/25	*Stenotrophomonas maltophilia*	Levofloxacin	≤ 0.5	Sensitive
			Trimetoprim-sulfametoxazole	≤ 2/38	Sensitive
PB Blood culture	04/21/25	*Stenotrophomonas maltophilia*	Levofloxacin	≤ 0.5	Sensitive
			Trimetoprim-sulfametoxazole	≤ 2/38	Sensitive
CVC Blood culture	04/21/25	*Stenotrophomonas maltophilia*	Levofloxacin	≤ 0.5	Sensitive
			Trimetoprim-sulfametoxazole	≤ 2/38	Sensitive

CVC: central venous catheter. MIC: minimum inhibitory concentration. PB: peripheral blood. Susceptibility interpretation was performed according to the current Clinical and Laboratory Standards Institute (CLSI) breakpoints.

Authorization was requested from the pharmacotherapeutic committee for the combined use of CZA with ATM, a regimen based on evidence of synergy against resistant strains of *S. maltophilia*. Treatment was started on day +32 (CZA 2 g every 8 h and ATM 2 g every 8 h, both in 3 h extended infusions). On that same day, given a positive serum galactomannan index (3.15), caspofungin was replaced by voriconazole, starting antifungal therapy for suspected invasive aspergillosis.

### Evolution

After starting the CZA + ATM combined treatment, along with levofloxacin and voriconazole and the replacement of the central venous catheter, the patient showed clinical improvement, with a progressive reduction in C-reactive protein (CRP) and resolution of the febrile clinical presentation. The blood culture from day +35 was negative, and a 14-day treatment cycle was completed (days +32 to +46). During this period, the patient remained clinically stable, with no signs of organ dysfunction or infection progression.

On day +49, he presented a new episode of persistent fever. Only the CZA + ATM regimen was empirically restarted, given the recent history of *S. maltophilia* bacteremia, and blood cultures were repeated. The new microbiological identification confirmed reinfection by *S. maltophilia*, this time with resistance to levofloxacin (MIC >1 µg/mL) (table 2). The CZA + ATM combination was maintained until day +64. From day +52, the patient remained afebrile, with a sustained decrease in CRP (peak of 18.6 mg/dL). From day +58, blood cultures were negative and remained so until day +90.

**Table 2:** Microbiological results of the second episode of bacteremia due to *Stenotrophomonas maltophilia*

Sample type	Date received	Isolate	Antimicrobial	MIC (µg/mL)	Interpretation
PB Blood culture	04/25/2025	*Stenotrophomonas maltophilia*	Levofloxacin	>1	Non-susceptible
			Trimethoprim-sulfametoxazole	≤2/38	Sensitive

MIC: minimum inhibitory concentration. PB: peripheral blood. Susceptibility interpretation was performed according to the Clinical and Laboratory Standards Institute (CLSI) criteria.

Throughout the follow-up, the patient presented profound pancitopenia, with persistent severe neutropenia (absolute neutrophil count <0.1 ×10⁹/L), anemia (Hb 7.0-8.2 g/dL), and thrombocytopenia (platelets <50 ×10⁹/L), with a nadir of 19 ×10⁹/L. Primary graft failure was confirmed, evidenced by the absence of hematopoietic recovery, CD4+ lymphopenia (<10%), and the absence of B lymphocytes and NK cells. Tacrolimus was suspended on day +21 due to lack of engraftment, and the patient was included on the waiting list for a second transplant. No new episodes of *S. maltophilia* bacteremia were documented until day +100 post-HSCT.

**Figure 1:**
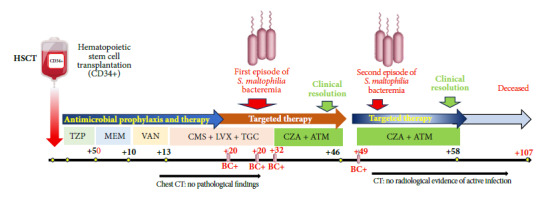
Chronological clinical course of a patient with recurrent S. maltophilia bacteremia after HSCT treated with ceftazidime-avibactam plus aztreonam.

HSCT, hematopoietic stem cell transplantation; BC, blood culture; TZP, piperacillin/tazobactam; MEM, meropenem; VAN, vancomycin; CMS, colistimethate sodium; LVX, levofloxacin; TGC, tigecycline; CZA, ceftazidime-avibactam; ATM, aztreonam; CT, computed tomography.

The patient died on day +107 due to septic shock in the context of profound neutropenia, associated with superimposed infections by *Pseudomonas aeruginosa* with a carbapenem-resistant phenotype, a VIM-type metallo-beta-lactamase producing strain (figure 1, supplementary material).

## DISCUSSION

*Stenotrophomonas maltophilia* is an emerging opportunistic pathogen of special relevance in immunocompromised patients, particularly in HSCT recipients. This case is clinically relevant due to the context of extreme immunological fragility, the presentation of two bacteremia episodes, and the need to use a non-conventional antimicrobial regimen due to local therapeutic limitations.

From a microbiological point of view, *S. maltophilia* presents a complex intrinsic resistance profile, primarily mediated by the production of chromosomal β-lactamases; L1, a metallo-β-lactamase capable of hydrolyzing penicillins, cephalosporins, and carbapenems, and L2, a serine β-lactamase that inactivates extended-spectrum cephalosporins and aztreonam ^14^. To this are added other mechanisms, such as biofilm formation, overexpression of efflux pumps, and outer membrane permeability alterations, which hinder the eradication of the microorganism and contribute to diagnostic uncertainty between colonization and active infection, particularly in immunosuppressed patients.

According to the practice guidelines issued by the Infectious Diseases Society of America (IDSA) in 2024, the treatment of invasive *S. maltophilia* infections lacks a clearly defined standard regimen and should be based on combination strategies. Proposed therapeutic options include trimethoprim-sulfamethoxazole, high-dose minocycline, fluoroquinolones (levofloxacin), and cefiderocol, preferably as part of combined regimens, due to the limited clinical evidence supporting monotherapy ^15^. However, these alternatives present significant limitations, particularly in immunocompromised patients, such as hematological toxicity, variability in bactericidal activity, risk of resistance selection during treatment, and scarce clinical experience in severe infections. In this sense, the combination of CZA plus ATM is recognized as a rational therapeutic strategy, as it overcomes the intrinsic resistance mechanisms mediated by L1 and L2 β-lactamases.

In the Peruvian context, applying these recommendations faces serious restrictions ^16^. Drugs such as cefiderocol and intravenous minocycline are unavailable. TMP-SMX, although effective in vitro, is contraindicated in patients with severe marrow aplasia due to its high risk of myelotoxicity ^15^. Levofloxacin, although accessible, lacks evaluation for its formal use in these infections by the Health Technology Assessment and Research Institute (IETSI) of the Social Health Insurance (EsSalud) in Peru ^17^.

Given these limitations, the patient’s treatment with the combination of ceftazidime-avibactam plus aztreonam, authorized under an alternative use protocol, allowed for complete clinical recovery and negative blood cultures within 72 hours. Subsequently, faced with a second episode with a levofloxacin-resistant strain (MIC >1 µg/mL), the exclusive reuse of the CZA + ATM combination was again effective. This evolution rules out the possible effect of levofloxacin administered in a previous regimen and underscores the value of this combination in multiple resistance scenarios.

From a microbiological approach, the synergy of this combination is based on the fact that avibactam inhibits the L2 β-lactamase, while aztreonam maintains its activity against the L1 metallo-β-lactamase ^18^. In recent preclinical models, Sangiorgio et al. evidenced that aztreonam/avibactam presents sustained activity against multidrug-resistant strains of *S. maltophilia*, supporting its potential clinical use in pulmonary infections ^19^.

The literature reports the successful use of the CZA with ATM combination in treating severe *S. maltophilia* infections ^20^, especially in multidrug-resistant strains ^7^ or in patients with contraindications to the use of trimethoprim-sulfamethoxazole ^11^. Lin et al. demonstrated, in an in vitro study, that the combination significantly reduced the minimum inhibitory concentrations (MIC) of aztreonam in the presence of avibactam, restoring sensitivity in more than 94% of clinical isolates ^13^. Vlaspolder et al. confirmed this synergy through optimized susceptibility methods, highlighting the joint effect of CZA and ATM against resistant strains ^21^. Likewise, a recent report published in 2024 documented the complete resolution of a case of post-neurosurgical meningitis due to multidrug-resistant *S. maltophilia* treated with CZA + ATM ^22^. Accordingly, Diarra et al. reported that the CZA and ATM combination successfully eliminated multidrug-resistant *S. maltophilia* bacteremia in an immunocompromised patient ^23^. However, to date, no specific reports have been described in HSCT patients; therefore, this case constitutes an original clinical contribution supporting the use of this combination in a context of severe immunosuppression and limited therapeutic options.

This case not only provides clinical evidence on a therapeutic alternative against *S. maltophilia* in the context of HSCT—a condition with high mortality if timely bacteriological control is not achieved—but also highlights the need to adapt international therapeutic guidelines to local realities. This could be achieved through the development of alternative use protocols and the establishment of antimicrobial evaluation committees.

Given the clinical complexities presented, the evaluation of new therapeutic regimens should be considered, especially those supported by evidence and adequate pharmacodynamic reasoning. In this sense, our findings suggest that the combination of CZA and ATM in post-HSCT patients with *S. maltophilia* bacteremia could represent a useful strategy in middle-income countries, at least while restrictions on access to innovative antimicrobials such as cefiderocol persist.

The present case report has some limitations that must be considered. First, molecular typing of the *S. maltophilia* isolates was not performed, preventing the certain determination of whether both episodes corresponded to recurrence or reinfection. Second, it involves the experience of a single patient, which limits the generalization of the results. Likewise, the coexistence of profound immunosuppression, primary graft failure, and multiple antimicrobial interventions makes it difficult to isolate the exclusive impact of the CZA and ATM combination on the clinical outcome. Nevertheless, the documented microbiological resolution in both episodes supports the clinical relevance of this therapeutic strategy in scenarios with limited options.

### Public Health Implications

In patients undergoing hematopoietic stem cell transplantation, continuous microbiological surveillance constitutes an essential component of clinical care, as it allows for the early detection of opportunistic pathogens, timely identification of changes in antimicrobial susceptibility profiles, and optimization of therapeutic decisions in contexts of profound immunosuppression. In countries like Peru, where access to latest-generation antimicrobials is limited and the availability of therapeutic options against multidrug-resistant pathogens is restricted, these surveillance systems acquire even greater relevance.

The present report provides local clinical evidence on the successful use of the CZA and ATM combination as a therapeutic alternative against *S. maltophilia* in a post-HSCT patient with a contraindication for the use of TMP-SMX. This finding is relevant for Peruvian public health, as it highlights the need to strengthen hospital microbiological surveillance programs, antimicrobial evaluation committees, and rational antimicrobial use strategies, particularly in high-complexity units such as hematology and transplant. Likewise, the case emphasizes the importance of generating national evidence that allows international recommendations to be adapted to local realities and contributes to the design of contextualized therapeutic protocols for highly vulnerable populations.

In conclusion, this case report demonstrates that the combination of ceftazidime-avibactam plus aztreonam was effective in achieving clinical and microbiological resolution of two episodes of *S. maltophilia* bacteremia in a patient undergoing allogeneic hematopoietic stem cell transplantation with a contraindication for the use of trimethoprim-sulfamethoxazole. The favorable response observed in both episodes, even against a second isolate with levofloxacin resistance, supports the clinical value of this therapeutic strategy in scenarios of profound immunosuppression and limited antimicrobial options.
