# Supplementary material for: Ceftazidime-avibactam and aztreonam as a therapeutic alternative in two episodes of bacteremia due to Stenotrophomonas maltophilia after allogeneic hematopoietic stem cell transplantation: case report
Source: Rev Peru Med Exp Salud Publica. 2026 Mar 2;43(1):126–31. doi: 10.17843/rpmesp.2026.431.15393. (PMC13245987; doi:10.17843/rpmesp.2026.431.15393.)
Supplement: Supplementary material. — Available in the electronic version of the RPMESP. [file rpmesp-43-01-15393-s001.docx]

**MATERIAL SUPLEMENTARIO**

**Tabla 1.** Cronología de eventos del paciente

| Día postinfusión | Evento clínico principal | Microbiología / Virología | Terapia / Intervención | Resultado clínico |
| --- | --- | --- | --- | --- |
| 0 | Infusión de células madre (CD34+) | — | Infusión de progenitores hematopoyéticos | Trasplante realizado |
| +1 a +4 | Postinfusión de ciclofosfamida | — | CTX IV días +1, +3 y +4 | Inmunosupresión postinfusión completada |
| +5 | Inicio de inmunosupresión de mantenimiento | — | TAC (con TDM) + MMF 15 mg/kg PO c/12 h | Estabilización inicial del paciente |
| +10 | Evaluación postinfusión | CMV VL: no detectable; GM sérico: negativo | — | Sin evidencia de infección |
| +20 | 1.ª bacteriemia | *S. maltophilia* en BC periférica y CVC (sensible a Lvx y TMP/SMX) | Retiro de CVC; no uso de TMP/SMX por mielosupresión | Resolución clínica inicial |
| +31 | Elevación de GM sérico | GM (R: 3.15) | — | Sin signos clínicos de aspergilosis |
| +33 | Evaluación virológica | EBV y ADV VL: no detectable | — | Sin evidencia de reactivación viral |
| +39 | Evaluación posterior | Hemocultivos periféricos y CVC: negativos | — | Sin bacteriemia |
| +40 | Reactivación viral | CMV VL: 4.8 log UI/mL; GM negativo | — | Estable, sin enfermedad clínica por CMV |
| +42 | Evaluación de infecciones fúngicas | HG (1.81); GM (R: 0.62) | — | Sin aspergilosis invasiva |
| +49 | 2.ª bacteriemia | *S. maltophilia* en BC periférica | Reinicio de CAZ/AVI + ATM | Control clínico logrado |
| +58 | Evaluación posterior | Cultivos periféricos y CVC: negativos; GM R: 0.42 | — | Sin bacteriemia ni signos de aspergilosis |
| +64 | Evaluación virológica | CMV VL: no detectable; GM R: 0.54 | — | Estable, sin infección activa |
| +106 | Evento final | *Pseudomonas aeruginosa* VIM y *Candida tropicalis* en cultivos finales | — | Fallecimiento por infección fúngico-bacteriana diseminada |

**Tabla 3.** Lista de verificación CARE

| **ítem** | **Descripción de cada elemento de la lista de verificación** | **Aplicado en el manuscrito** |
| --- | --- | --- |
| 1 | Diagnóstico/intervención principal con 'case report' | ✔ |
| 2 | Palabras clave (2 a 5) | ✔ |
| 3a | Introducción: ¿Qué lo hace único? | ✔ |
| 3b | Síntomas y hallazgos clínicos | ✔ |
| 3c | Diagnóstico, tratamiento, resultado | ✔ |
| 3d | Conclusión | ✔ |
| 4 | Resumen del por qué es único | ✔ |
| 5a | Información del paciente no identificada | ✔ |
| 5b | Síntomas principales | ✔ |
| 5c | Historia médica, familiar, psicosocial | ✔ |
| 5d | Intervenciones previas relevantes | ✔ |
| 6 | Hallazgos físicos importantes | ✔ |
| 7 | Cronología en forma de timeline | ✔ |
| 8a | Pruebas diagnósticas | ✔ |
| 8b | Desafíos diagnósticos | ✔ |
| 8c | Diagnóstico (otros considerados) | ✔ |
| 8d | Pronóstico | ✔ |
| 9a | Tipo de intervención terapéutica | ✔ |
| 9b | Administración del tratamiento | ✔ |
| 9c | Cambios en el tratamiento | ✔ |
| 10a | Resultados clínicos del paciente | ✔ |
| 10b | Resultados de seguimiento | ✔ |
| 10c | Adherencia y tolerabilidad | ✔ |
| 10d | Eventos adversos no anticipados | ✔ |
| 11a | Discusión de fortalezas y limitaciones | ✔ |
| 11b | Revisión de literatura con referencias | ✔ |
| 11c | Razonamiento científico para conclusiones | ✔ |
| 11d | Lección principal del caso | ✔ |
| 12 | Perspectiva del paciente | ✘ (Falleció) |
| 13 | Consentimiento informado | ✔ |
