# Supplementary material for: Ceftazidime-avibactam and aztreonam as a therapeutic alternative in two episodes of bacteremia due to Stenotrophomonas maltophilia after allogeneic hematopoietic stem cell transplantation: case report
Source: Rev Peru Med Exp Salud Publica. 2026 Mar 2;43(1):126–31. doi: 10.17843/rpmesp.2026.431.15393. (PMC13245987; doi:10.17843/rpmesp.2026.431.15393.)
Supplement: Translation

# Translation: Ceftazidima-avibactam y aztreonam como alternativa terapéutica en dos episodios de bacteriemia por *Stenotrophomonas maltophilia* postrasplante alogénico de progenitores hematopoyéticos: reporte de caso

**Authors:** Wilder Bolaños-Vargas, Cesar Copaja-Corzo, Ivan Fernandez Vertiz, Alfredo Wong-Chang, Miriam Rut Baldeon Laureano

**Affiliations:** 1 Departamento de Farmacia, Hospital Nacional Edgardo Rebagliati Martins, Lima, Perú.; 2 Unidad de Investigación para la Generación y Síntesis de Evidencias en Salud, Universidad San Ignacio de Loyola, Lima, Perú.; 3 Servicio de Infectología, Hospital Nacional Edgardo Rebagliati Martins, Lima, Perú.; 4 Departamento de Hematología, Servicio de Hematología Especial, Hospital Nacional Edgardo Rebagliati Martis, Lima, Perú.; 5 Hospital Nacional Edgardo Rebagliati Martins, Lima, Peru.

**Keywords:** Stenotrophomonas maltophilia, trasplante de progenitores hematopoyéticos, ceftazidima-avibactam, aztreonam, neutropenia febril

## Abstract

La infección por *Stenotrophomonas maltophilia* representa un desafío terapéutico en pacientes inmunocomprometidos, especialmente durante la recuperación hematológica postrasplante de progenitores hematopoyéticos (TPH). El uso de trimetoprim-sulfametoxazol (TMP-SMX), antimicrobiano de primera línea, se encuentra contraindicado por su toxicidad hematológica. Presentamos el caso de un paciente sometido a TPH haploidéntico que desarrollo dos episodios de bacteriemia por *S. maltophilia* en los días +20 y +49. Ante la contraindicación de TMP-SMX por mielosupresión severa, se empleó la combinación de ceftazidima-avibactam y aztreonam como tratamiento alternativo, logrando resolución clínica en ambos episodios. El paciente permaneció estable hasta el día +100; Sin embargo, presentó fracaso del injerto y falleció en el día +107 por infección por *Pseudomonas aeruginosa* productora de carbapenemasa tipo VIM. Este caso muestra que el uso de ceftazidima-avibactam más aztreonam puede ser de utilidad frente a infecciones por *S. maltophilia* en pacientes pos-TPH con contraindicación para el uso de TMP-SMX.

## INTRODUCCIÓN

*Stenotrophomonas maltophilia* (*S. maltophilia*) es un bacilo gramnegativo no fermentador reconocido como un patógeno oportunista emergente, particularmente en pacientes inmunocomprometidos, como aquellos sometidos a trasplante alogénico de progenitores hematopoyéticos (TPH*)*^1^. En este contexto, la inmunosupresión profunda y sostenida favorece tanto a la colonización como a la infección por microorganismos ambientales como *S. maltophilia*, capaz de producir infecciones invasivas graves, incluidas neumonía, bacteriemia persistente y sepsis ^2^^,^^3^. Su presencia se asocia con una alta morbimortalidad en este grupo de pacientes, lo que subraya la importancia de estrategias terapéuticas efectivas ^4^.

Uno de los principales desafíos en el tratamiento de infecciones por *S. maltophilia* radica en su perfil de resistencia antimicrobiana intrínseca, mediada por múltiples mecanismos como bombas de eflujo, alteración en la permeabilidad de la membrana externa y la producción de dos β-lactamasas cromosómicas ^5^. La β-lactamasa L1, es una metalobetalactamasa (MBL) que hidroliza penicilinas, cefalosporinas y carbapenémicos, aunque no afecta al aztreonam. Por su parte, la L2, es una serina β-lactamasa con capacidad de inactivar cefalosporinas de espectro extendido y aztreonam, pero suceptible a inhibicion por avibactam ^6^. Esta combinacion de mecanismos reduce significativamente la actividad de la mayoría de los β-lactámicos convencionales frente a este patógeno ^7^. A pesar de ello, trimetoprim-sulfametoxazol (TMP-SMX) continúa siendo el tratamiento de elección ^8^.

Sin embargo, en pacientes trasplantados con mielosupresión grave, el uso de TMP-SMX puede estar contraindicado debido a su toxicidad hematológica, incluyendo neutropenia y trombocitopenia. En tales escenarios clínicos, se vuelve necesario recurrir a terapias alternativas que preserven la actividad antimicrobiana sin comprometer la recuperación hematopoyética ^9^^,^^10^. La combinación de ceftazidima-avibactam (CZA) con aztreonam (ATM) ha surgido como una estrategia terapéutica prometedora frente a *S. maltophilia*, particularmente en contextos de resistencia o contraindicación al TMP-SMX ^11^. Esta combinación actúa de manera sinérgica sobre los mecanismos enzimáticos de resistencia intrínsecos de la bacteria ^12^. Aunque los datos clínicos sobre el uso combinado de CZA y ATM aún son limitados y provienen mayoritariamente en estudios observacionales y reportes de caso, la evidencia *in vitro* indica que la formulación mimética aztreonam-avibactam exhibe actividad frente a aproximadamente el 92% de los aislamientos de *S. maltophilia*^13^. Por ello, esta combinación se considera una opción terapéutica válida en escenarios clínicos complejos, a pesar de la falta de estudios controlados.

En este contexto, se presenta el caso de un paciente sometido a TPH, con contraindicación absoluta para TMP-SMX, que desarrolló dos episodios de bacteriemia por *S. maltophilia* y fue tratado exitosamente con la combinación de CZA y ATM. El objetivo de este reporte es proporcionar evidencia clínica que demuestre la necesidad de evaluar el uso de CZA y ATM como alternativa terapéutica en pacientes pos-TPH durante la fase de mayor supresión medular (nadir) y prendimiento hematopoyético, un escenario caracterizado por inmunosupresión severa y opciones antimicrobianas limitadas frente a *S. maltophilia*.

## CASO CLÍNICO

Varón de 36 años con diagnóstico de síndrome mielodisplásico (SMD) con fibrosis medular asociada, clasificado como de riesgo intermedio según el puntaje del International Prognostic Scoring System - Revised (IPSS-R) ^3^. Debido a su requerimiento transfusional crónico y la ausencia de un donante HLA idéntico, se decidió realizar un TPH haploidéntico con acondicionamiento mieloablativo. El esquema de acondicionamiento incluyó busulfán intravenoso con un área bajo la curva (AUC_24h_) objetivo de 4500 µmol·min y fludarabina con AUC_24h_ objetivo de 5 mg·h/L (administrada entre los días -6 y -2). El día 0 se realizó la infusión de células madre hematopoyéticas (CD34+), seguida de profilaxis de enfermedad injerto contra huésped (EICH) con ciclofosfamida pos-TPH (50 mg/kg los días +3 y +4), y posteriormente tacrolimus (0.075 mg/kg) más micofenolato mofetilo (15 mg/kg/día) desde el día +5 hasta el día +180.

### Intervención

Desde el día +1 postrasplante, el paciente presentó fiebre persistente (>38,3 °C) sin foco clínico aparente. Se inició tratamiento empírico con piperacilina-tazobactam (4,5 g IV cada 6 h). Ante la ausencia de mejoría, el esquema se escaló a meropenem (2 g IV cada 8 h en infusión extendida de 3 horas) desde el día +5. La persistencia de la fiebre motivó la adición de vancomicina a partir del día +10, con ajuste individualizado mediante modelado bayesiano para alcanzar un AUC/CIM entre 400-600.

Entre los días +11 y +14, múltiples hemocultivos tanto periféricos como del catéter resultaron negativos. En el contexto de neutropenia profunda y prolongada, inmunosupresión intensa y falta de respuesta al esquema antibiótico de amplio espectro, se sospechó infección fúngica invasiva (IFI). Por ello, se instauró tratamiento antifúngico empírico con caspofungina desde el día +13 (dosis de carga de 70 mg seguida de 50 mg/día).

El día +20, el paciente persistía febril, pese a encontrarse en tratamiento con meropenem, vancomicina y caspofungina. Se sospechó infección por bacilos gramnegativos multirresistentes, por lo que se añadió cobertura con levofloxacino (750 mg VO cada 24 h) y colistina (dosis de carga de 300 mg seguida de 150 mg IV cada 12 h) y se repitieron los hemocultivos. El día +23, se reportó el crecimiento de bacilos gramnegativos (hemocultivo de catéter venoso central [CVC] y periférico tomado el día +20), y ante la ausencia de mejoría clínica, se suspendieron meropenem y vancomicina el día +25 e inició tigeciclina 100 mg cada 12h como parte de una estrategia escalonada frente a patógenos multirresistentes.

El día +28 se confirmó la presencia de *S. maltophilia* en hemocultivos del CVC y periféricos tomados el día +20, con sensibilidad a levofloxacino y TMP-SMX (tabla 1). Sin embargo, se descartó el uso de TMP/SMX por su potencial mielotoxicidad, considerando el estado de aplasia medular del paciente y la falta de prendimiento del trasplante.

**Tabla 1:** Resultados microbiológicos del primer episodio de bacteriemia por *Stenotrophomonas maltophilia*

Tipo de muestra	Fecha de recepción	Aislamiento	Antimicrobiano	CIM (µg/mL)	Interpretación
Hemocultivo CVC	12-04-25	*Stenotrophomonas maltophilia*	Levofloxacina	≤ 0,5	Sensible
			Trimetoprim-sulfametoxazol	≤ 2/38	Sensible
Hemocultivo CVC	10-04-25	*Stenotrophomonas maltophilia*	Levofloxacina	≤ 0,5	Sensible
			Trimetoprim-sulfametoxazol	≤ 2/38	Sensible
Hemocultivo SP	14-04-25	*Stenotrophomonas maltophilia*	Levofloxacina	≤ 0,5	Sensible
			Trimetoprim-sulfametoxazol	≤ 2/38	Sensible
Hemocultivo CVC	14-04-25	*Stenotrophomonas maltophilia*	Levofloxacina	≤ 0,5	Sensible
			Trimetoprim-sulfametoxazol	≤ 2/38	Sensible
Hemocultivo SP	21-04-25	*Stenotrophomonas maltophilia*	Levofloxacina	≤ 0,5	Sensible
			Trimetoprim-sulfametoxazol	≤ 2/38	Sensible
Hemocultivo CVC	21-04-25	*Stenotrophomonas maltophilia*	Levofloxacina	≤ 0,5	Sensible
			Trimetoprim-sulfametoxazol	≤ 2/38	Sensible

CVC: catéter venoso central. CIM: concentración inhibitoria mínima. SP: sangre periférica. La interpretación de susceptibilidad se realizó de acuerdo con los puntos de corte del Clinical and Laboratory Standards Institute (CLSI) vigentes.

Se solicitó autorización al comité farmacoterapéutico para el uso combinado de CZA con ATM, esquema basado en evidencia de sinergia frente a cepas resistentes de *S. maltophilia*. El tratamiento se inició el día +32 (CZA 2 g cada 8 h y ATM 2 g cada 8 h, ambos en infusión extendida de 3 h). Ese mismo día, ante un índice de galactomanano sérico positivo (3,15), se sustituyó caspofungina por voriconazol, iniciando terapia antifúngica por sospecha de aspergilosis invasiva*.*

### Evolución

Tras el inicio del tratamiento combinado CZA + ATM, junto con levofloxacino y voriconazol y el recambio del catéter venoso central, el paciente evidenció mejoría clínica, con reducción progresiva de la proteína C reactiva (PCR) y resolución del cuadro febril. El hemocultivo del día +35 fue negativo, y se completó un ciclo de 14 días de tratamiento (días +32 a +46). Durante este periodo, el paciente permaneció clínicamente estable, sin signos de disfunción orgánica ni progresión de la infección.

El día +49, presentó un nuevo episodio de fiebre persistente. Se reinició empíricamente solo el esquema CZA + ATM, dada la historia reciente de bacteriemia por *S. maltophilia*, y se repitieron hemocultivos. La nueva identificación microbiológica confirmó reinfección por *S. maltophilia*, esta vez con resistencia a levofloxacino (CIM >1 µg/mL) (tabla 2). Se mantuvo la combinación CZA + ATM hasta el día +64. Desde el día +52, el paciente permaneció afebril, con descenso sostenido de la PCR (pico de 18,6 mg/dL). A partir del día +58, los hemocultivos fueron negativos y permanecieron así hasta el día +90.

**Tabla 2:** Resultados microbiológicos del segundo episodio de bacteriemia por *Stenotrophomonas maltophilia.*

Tipo de muestra	Fecha de recepción	Aislamiento	Antimicrobiano	CIM (µg/mL)	Interpretación
Hemocultivo SP	25-04-2025	*Stenotrophomonas maltophilia*	Levofloxacino	>1	No sensible
			Trimetoprim-sulfametoxazol	≤2/38	Sensible

CIM: concentración inhibitoria mínima. SP: sangre periférica. La interpretación de susceptibilidad se realizó de acuerdo con los criterios del Clinical and Laboratory Standards Institute (CLSI).

Durante todo el seguimiento, el paciente presentó pancitopenia profunda, con neutropenia severa persistente (recuento absoluto de neutrófilos <0,1 ×10⁹/L), anemia (Hb 7,0-8.2 g/dL) y trombocitopenia (plaquetas <50 ×10⁹/L), con nadir de 19 ×10⁹/L. Se confirmó fracaso primario del injerto, evidenciado por ausencia de recuperación hematopoyética, linfopenia CD4+ (<10%), ausencia de linfocitos B y células NK. Tacrolimus fue suspendido el día +21 por falta de prendimiento, y el paciente fue incluido en lista de espera para un segundo trasplante. Hasta el día +100 pos-TPH no se documentaron nuevos episodios de bacteriemia por *S. maltophilia*.

El paciente falleció el día +107 debido a shock séptico en el contexto de una neutropenia profunda, asociado a infecciones sobreagregadas por *Pseudomonas aeruginosa* con fenotipo de resistencia a carbapenémicos cepa productora de metalobetalactamasas de tipo VIM (figura 1).

**Figura 1:** Curso clínico cronológico de paciente con bacteriemia recurrente por *S. maltophilia* tras TPH tratado con ceftazidima-avibactam más Aztreonam.

TPH, trasplante de progenitores hematopoyéticos; HC, hemocultivo; TZP, piperacilina/tazobactam; MEM, meropenem; VAN, vancomicina; CMS, colistimetato de sodio; LVX, levofloxacino; TGC, tigeciclina; CZA, ceftazidima-avibactam; ATM, aztreonam; TC, tomografía computarizada.

## DISCUSIÓN

*Stenotrophomonas maltophilia* es un patógeno oportunista emergente de especial relevancia en pacientes inmunocomprometidos, particularmente en receptores de TPH. Este caso resulta clínicamente relevante por el contexto de extrema fragilidad inmunológica, la presentación de dos episodios de bacteremia, y la necesidad de utilizar un régimen antimicrobiano no convencional debido a las limitaciones terapéuticas del entorno local*.*

Desde el punto de vista microbiológico*, S. maltophilia* presenta un perfil de resistencia intrínseca complejo, mediado principalmente por la producción de β-lactamasas cromosómicas; L1 una metalo-β-lactamasa capaz de hidrolizar penicilinas cefalosporinas y carbapenémicos, y L2, una serina β-lactamasa que inactiva cefalosporinas de espectro extendido y aztreonam ^14^, a ello se suman otros mecanismos, como la formulación de biopelículas, la sobreexpresión de bombas de eflujo y alteraciones de la permeabilidad de la membrana externa, que dificulta la erradicación del microorganismo y contribuyen a la incertidumbre diagnostica entre colonización e infección activa, particularmente en pacientes inmunosuprimidos.

Según la guía de práctica emitida *por la* Infectious Diseasess Society of America (IDSA) en 2024, el tratamiento de las infecciones invasivas por *S. maltophilia* carece de un esquema estándar claramente definido y debe basarse en estrategias combinadas. Las opciones terapéuticas propuestas incluyen trimetropim-sulfametaxazol, minociclina en dosis altas, fluoroquinolonas (levofloxacino) y cefiderocol, preferentemente como parte de esquemas combinados, debido a la limitada evidencia clínica que respalde la monoterapia ^15^. No obstante, esas alternativas presentan importantes limitaciones, particularmente en pacientes inmunocomprometidos, como toxicidad hematológica, variabilidad en la actividad bactericida, riesgo de selección de resistencia durante el tratamiento y escasa experiencia clínica en infecciones graves. En ese sentido, la combinación de CZA más ATM es reconocida como una estrategia terapéutica racional, al permitir superar los mecanismos de resistencia intrínseca de mediados por las β-lactamasas L1 y L2.

En el contexto peruano la aplicación de estas recomendaciones enfrenta serias restricciones ^16^. Fármacos como cefiderocol y minociclina intravenosa no se encuentran disponibles. TMP-SMX, aunque eficaz *in vitro*, está contraindicado en pacientes con aplasia medular severa, por su elevado riesgo de mielotoxicidad ^15^. Levofloxacino, aunque accesible, carece de evaluación para su uso formal en estas infecciones por parte del Instituto de Evaluación de Tecnologías en Salud e Investigación (IETSI) del Seguro Social de Salud (EsSalud) en Perú ^17^.

Ante estas limitaciones, el tratamiento del paciente con la combinación de ceftazidima-avibactam más aztreonam, autorizado bajo un protocolo de uso alternativo, permitió una recuperación clínica completa y negativización de hemocultivos a las 72 horas. Posteriormente, frente a un segundo episodio con una cepa resistente a levofloxacino (CIM >1 µg/mL), la reutilización exclusiva de la combinación CZA + ATM volvió a ser eficaz. Esta evolución descarta el posible efecto del levofloxacino administrado en un esquema previo y subraya el valor de esta combinación en escenarios de resistencia múltiple.

Desde un enfoque microbiológico, la sinergia de esta combinación se fundamenta en que avibactam inhibe la β-lactamasa L2, mientras que aztreonam mantiene su actividad frente a la metalo-β-lactamasa L1 ^(^^18^. En modelos preclínicos recientes, Sangiorgio *et al*. evidenciaron que aztreonam/avibactam presenta actividad sostenida contra cepas multirresistentes de *S. maltophilia*, respaldando su potencial uso clínico en infecciones pulmonares ^19^.

La literatura reporta el uso exitoso de la combinación CZA con ATM en el tratamiento de infecciones graves por *S. maltophilia*^20^, especialmente en cepas multirresistentes ^7^ o en pacientes con contraindicación al uso de trimetoprima-sulfametoxazol ^11^. Lin *et al*. demostraron, en un estudio *in vitro*, que la combinación redujo significativamente las concentraciones inhibitorias mínimas (CIM) de aztreonam en presencia de avibactam, restaurando la sensibilidad en más del 94 % de los aislamientos clínicos ^13^. Vlaspolder *et al*. confirmaron esta sinergia mediante métodos optimizados de susceptibilidad, destacando el efecto conjunto de CZA y ATM frente a cepas resistentes ^21^. Así mismo, y un reporte reciente publicado en 2024 documentó la resolución completa de un caso de meningitis posneuroquirúrgica por *S. maltophilia* multirresistente tratado con CZA + ATM ^22^. De manera concordante, Diarra *et al*, reportaron que la combinación de CZA y ATM logró eliminar la bacteriemia por *S. maltophilia* multirresistente en una paciente inmunocomprometida ^23^. No obstante, hasta la fecha no se han descrito reportes específicos en pacientes sometidos a TPH, por lo que el presente caso constituye una contribución clínica original que respalda el uso de esta combinación en un contexto de inmunosupresión severa y opciones terapéuticas limitadas.

Este caso no solo aporta evidencia clínica sobre una alternativa terapéutica contra *S. maltophilia* en el contexto de TPH, una condición de alta mortalidad si no se logra un control bacteriológico oportuno, sino que también resalta la necesidad de adaptar las guías terapéuticas internacionales a las realidades locales. Esto podría lograrse mediante el desarrollo de protocolos de uso alternativo y la conformación de comités de evaluación antimicrobiana.

Dadas las complejidades clínicas presentadas, se debe considerar la evaluación de nuevos esquemas terapéuticos, sobre todo aquellos respaldados por evidencia y razonamiento farmacodinámico adecuado. En este sentido, nuestros hallazgos sugieren que la combinación de CZA y ATM en pacientes post TPH y bacteriemia por *S. maltophilia* podría representar una estrategia útil en países de ingresos medios, al menos mientras persistan las restricciones de acceso a antimicrobianos innovadores como cefiderocol.

El presente reporte de caso presenta algunas limitaciones que deben ser consideradas. En primer lugar, no se realizó tipificación molecular de los aislamientos de *S. maltophilia,* lo que impide establecer con certeza si ambos episodios correspondieron a recurrencia o reinfección. En segundo lugar, se trata de la experiencia de un único paciente, lo que limita la generalización de los resultados. Asimismo, la coexistencia de inmunosupresión profunda, fracaso primario del injerto y múltiples intervenciones antimicrobianas dificulta aislar el impacto exclusivo de la combinación CZA y ATM sobre el desenlace clínico. No obstante, la resolución microbiológica documentada en ambos episodios respalda la relevancia clínica de esta estrategia terapéutica en escenarios de opciones limitadas.

### Implicancias para la salud pública

En pacientes sometidos a trasplante de progenitores hematopoyéticos, la vigilancia microbiológica continua constituye un componente esencial de la atención clínica, ya que permite la detección temprana de patógenos oportunistas, la identificación oportuna de cambios en los perfiles de susceptibilidad antimicrobiana y la optimización de las decisiones terapéuticas en contextos de inmunosupresión profunda. En países como el Perú, donde el acceso a antimicrobianos de última generación es limitado y la disponibilidad de opciones terapéuticas frente a patógenos multirresistentes es restringida, estos sistemas de vigilancia adquieren una relevancia aún mayor.

El presente reporte aporta evidencia clínica local sobre el uso exitoso de la combinación CZA y ATM como alternativa terapéutica frente *a S. maltophilia* en un paciente pos-TPH con contraindicación para el uso de TMP-SMX. Este hallazgo resulta relevante para la salud pública peruana, ya que evidencia la necesidad de fortalecer programas de vigilancia microbiológica hospitalaria, comités de evaluación antimicrobiana y estrategias de uso racional de antimicrobianos, particularmente en unidades de alta complejidad como hematología y trasplante. Asimismo, el caso resalta la importancia de generar evidencia nacional que permita adaptar las recomendaciones internacionales a las realidades locales y contribuir al diseño de protocolos terapéuticos contextualizados para poblaciones altamente vulnerables.

En conclusión, este reporte de caso demuestra que la combinación de ceftazidima-avibactam más aztreonam fue eficaz para lograr la resolución clínica y microbiológica de dos episodios de bacteriemia por *S. maltophilia* en un paciente sometido a trasplante alogénico de progenitores hematopoyéticos con contraindicación para el uso de trimetoprim-sulfametoxazol. La respuesta favorable observada en ambos episodios, incluso frente a un segundo aislamiento con resistencia a levofloxacino, respalda el valor clínico de esta estrategia terapéutica en escenarios de inmunosupresión profunda y opciones antimicrobianas limitadas.
